# Optimization of a Method for Extraction and Determination of Residues of Selected Antimicrobials in Soil and Plant Samples Using HPLC-UV-MS/MS

**DOI:** 10.3390/ijerph18031159

**Published:** 2021-01-28

**Authors:** Klaudia Kokoszka, Agnieszka Kobus, Sylwia Bajkacz

**Affiliations:** Department of Inorganic Chemistry, Analytical Chemistry and Electrochemistry, Faculty of Chemistry, Silesian University of Technology, 44-100 Gliwice, Poland; klaudia.kokoszka@polsl.pl (K.K.); agagrabiec1@wp.pl (A.K.)

**Keywords:** emerging pollutants, pharmaceuticals, target analysis, environmental samples, antimicrobials accumulation

## Abstract

The residues of antimicrobials used in human and veterinary medicine are popular pollutants of anthropogenic origin. The main sources of introducing antimicrobials into the environment are sewage treatment plants and the agricultural industry. Antimicrobials in animal manure contaminate the surrounding soil as well as groundwater, and can be absorbed by plants. The presence of antimicrobials in food of plant origin may pose a threat to human health due to their high biological activity. As part of the research, a procedure was developed for the extraction and determination of ciprofloxacin, enrofloxacin, cefuroxime, nalidixic acid and metronidazole in environmental samples (soil and parsley root). An optimized solid-liquid extraction (SLE) method was used to separate antimicrobials from the solid samples and a mixture of citrate buffer (pH = 4): methanol (1:1; *v*/*v*) was used as the extraction solvent. Solid phase extraction (SPE) with OASIS^®^ HLB cartridges was used to purify and pre-concentrate the sample. The recovery of the developed method was in the range of 55–108%. Analytes were determined by high-performance liquid chromatography coupled with an ultraviolet (UV) detector and a tandem mass spectrometer (HPLC-UV-MS/MS). The procedure was validated and applied to the determination of selected antimicrobials in soil and parsley root samples. Five types of soil and five types of parsley roots of different origins were analyzed. The presence of nalidixic acid in the parsley root samples was found in the concentration range of 0.14–0.72 ng g^−1^. It has been shown that antimicrobials are absorbed by the plant and can accumulate antimicrobials in its edible parts.

## 1. Introduction

The progress of civilization strongly depends on constant access to pharmaceuticals. In highly developed countries, pharmaceutical residues are a common type of soil [1] and water pollution [2,3]. Antibiotics are a particularly dangerous group of pollutants (referred to as new emerging pollutants) due to their high biological activity at low concentrations, a tendency to accumulate in the environment and often high stability to abiotic and biotic degradation used in treatment processes [4,5]. In 2015, the total consumption of antibiotics in Europe ranged from 3.55 to 1195.69 tons per year, and in Poland alone it was 306.61 tons per year [6]. High consumption of antibiotics in Poland according to the report of the Supreme Audit Office no. 40/2019/P/18/058/KZD results from an improperly functioning health care system. It has been shown that antibiotics are prescribed by doctors without microbiological diagnosis [7].

The main recipients of antibiotics are the medical and veterinary industries. Antibiotics are used as feed additives in pig, cattle and poultry farms to increase meat production, treat and prevent herd diseases [8]. The use of pharmaceuticals for preventive purposes is prohibited in the European Union under Directive 2001/82/EC, however, this practice is still carried out. The main source of antibiotics introduced into the environment are human and animal excrement. Antibiotics, along with the faeces, go to a sewage treatment plant where they should be mineralized. However, antibiotics may be stable under the conditions of microbial cleansing and chlorination, or they may undergo transformations leading to the formation of toxic transformation products [4,9].

Wastewater treatment plants are the main source of water pollution due to the presence of pharmaceutical residues in treated water discharged into rivers [10]. The effluents from wastewater treatment plants may contain 28.4–584.9 ng L^−1^ ciprofloxacin (CIP), 10.1–69.4 ng L^−1^ enrofloxacin (ENF), 15.6–50.3 ng L^−1^ nalidixic acid (NAL) and 5.31–88.6 ng L^−1^ of metronidazole (MET) [11]. Cefuroxime is also detected in the effluents from wastewater treatment plants and its content is in the range of 3.43–600 ng L^−1^ [12,13].

The highest concentrations of fluoroquinolones (FQs) are found in the excrement of pigs and chickens, which are the most popular livestock [14]. ENF and its active metabolite CIP are commonly detected in poultry and pig manure and in the soil fertilized with it [15,16]. The content of FQs in manure samples may be in the range for ENF 3.0–7000 and CIP 1.0–520.0 µg kg^−1^, and for soil fertilized with manure CIP 5.8–17.1 and ENF 2.5–20.6 µg kg^−1^ [17,18]. Fluoroquinolones are stable in the composting process, and their content in commercially used manure after composting may range from 17.8–2395 µg kg^−1^ CIP and 8.8–1540 µg kg^−1^ ENF [19]. In manure fertilized soils, selected antibiotics may persist for up to 5 months after application [20]. In our previous study, we determined the content of antibiotics in the samples of activated sludge and manure after anaerobic digestion [21]. It was observed that the samples contained the residues of amoxicillin, ampicillin, metronidazole, sulfamethoxazole and cefuroxime.

The negative effect of the accumulation of antibiotics in the environment is that non-target organisms are exposed to them. The most common example of the negative impact on organisms is the phenomenon of drug resistance [22].

FQs present in the soil may have a strong phytotoxic effect and disrupt the plant’s hormonal balance [23]. ENF in the concentration range of 0.5–50 mM inhibits plant germination by 10–95%, while at concentrations of 0.5–10 mM it inhibits the elongation growth of the roots and stem of the plant [24]. CIP is present in the soil in concentrations of 6–10 mg kg^−1^ during growth and development of carrots and is detected in higher concentrations in the roots than in the leaves [25]. Antibiotics are also present in edible parts of plants, which can be dangerous to human health. CIP and MET were detected in lettuce leaves sold in Ghana and their concentrations ranged from 10.0–50.0 ng kg^−1^ [26]. Model experiments carried out in greenhouse conditions showed that ENF can accumulate in radish tissues 45 days after sowing. The concentration of ENF in radish tissues is lower than 0.02 mg kg^−1^, for the initial concentration of the antibiotic in the soil of between 5.0 and 20.0 mg kg^−1^ [27].

A wide variety of techniques for the extraction of antibiotics from solid samples are described in the literature. The most commonly used are solid-liquid extraction (SLE) [25], ultrasound assisted extraction (UAE) [28] and pressurized liquid extraction (PLE) [29,30] combined with sample purification and pre-concentration using solid phase extraction (SPE) [17,31,32,33,34]. High-performance liquid chromatography combined with a tandem mass spectrometer (HPLC-MS/MS) [17,32,33,35] is most often used to determine selected analytes in environmental samples, due to its high sensitivity and selectivity. The use of ultraviolet (UV) and fluorimetric detectors for the analysis of environmental samples is much less common, due to the low concentration range of antibiotics in the environment [36,37].

The aim of the research was to select the conditions for the extraction and determination of three quinolone antibiotics (CIP, ENF, NAL), cefuroxime (CEF) and metronidazole (MET) from solid environmental samples. A procedure for isolating the selected antimicrobials from parsley root and soil samples was developed and optimized using the combined SLE-SPE techniques. The determination of antimicrobials was conducted using HPLC-UV-MS/MS. After validation the procedure was used to analyse selected drugs in parsley root and soil and the results obtained indicate the presence of NAL in some of the parsley samples.

## 2. Materials and Methods

### 2.1. Standard, Chemicals and Materials

Analytical standards of ciprofloxacin, enrofloxacin, nalidixic acid, metronidazole and cefuroxime (purity of all >98%) were purchased from Sigma-Aldrich (St. Louis, MO, USA) (Table 1). Analytical grade acetonitrile, methanol, acetone, ethyl acetate and hydrochloric acid were purchased from Chempur (Piekary Śląskie, Poland). Analytical grade acetic acid was purchased from POCH S.A (Gliwice, Poland). Analytical grade ammonium acetate and citrate buffer (pH = 2, pH = 3, pH = 4, pH = 5, pH = 6) were purchased from Merck (Darmstadt, Germany). Hypergrade water, methanol, acetonitrile and formic acid were purchased from Merck (Darmstadt, Germany).

Oasis HLB cartridges (500 mg, 6 mL) from Waters (Eschborn, Germany) were used for solid-phase extraction (SPE). Nylon syringe filters (0.45 µm, 25 mm, PURELAND) were used to filter the sample. Plant material samples (parsley root) were purchased from stores in the Silesian Voivodship (administrative unit) in Poland. Soil samples were collected in Gliwice, Poland.

### 2.2. Preparation of Standard Solution

Standard solutions of ENF, CEF, NAL, CIP and MET with a concentration of 1 mg mL^−1^ in methanol were prepared. The addition of 10 µL acetic acid to 1.0 mL methanol was required to completely dissolve CIP. Working solutions of the selected analytes with concentrations of 100, 500 and 1000 ng mL^−1^ were prepared by diluting the standard solution. The plant material and soil was then enriched with a mixture of antimicrobials at three concentration levels: low (LQC) 1 ng g^−1^, medium 80 ng g^−1^ (MQC) and high 160 ng g^−1^ (HQC). Standard and working solutions of antimicrobials were stored in the dark at 4 °C.

### 2.3. Sample Preparation

The procedure for extracting pharmaceuticals was developed using the parsley root and soil samples without selected antimicrobials, which were blank samples. The parsley root was bought at a local store and then air-dried and ground in an electric grinder. The soil samples were dried and then sieved through a metal sieve with a diameter of 0.6 mm.

After drying, soil and parsley root samples were kept inside polyethylene bags and storage at 4 °C during 21 days.

One gram of the prepared samples was weighed and enriched with a mixture of antimicrobials of a defined concentration (5 µg g^−1^). As part of the optimization, parameters such as the type of solvent (1: methanol; 2: methanol:acetonitrile (1:1; *v*/*v*); 3: methanol:0.1 M HCl (1:1; *v*/*v*); 4: acetonitrile:acetone (1:1; *v*/*v*); 5: acetonitrile:ethyl acetate (1:1; *v*/*v*); 6: acetonitrile:0.01 M HCl (1:1; *v*/*v*); 7: 2% acetic acid in methanol:acetonitrile (1:1; *v*/*v*); 8: citrate buffer (pH = 4):methanol (1:1; *v*/*v*); 9: citrate buffer (pH = 4):acetonitrile (1:1; *v*/*v*)), the ratio of the mass of the sample to the volume of the solvent (from 1:10 to 1:40), shaking time (from 20 min to 120 min) and extraction intensity (750 rpm and 900 rpm), the effect of ultrasounds on the SLE extraction efficiency were checked. The best procedure was selected based on the recovery value. HPLC-UV was used for the analysis of extracts during optimization of the extraction procedure.

In an optimized extraction procedure, 20 mL of a mixture of citrate buffer (pH = 4):methanol (1:1; *v*/*v*) was added to 1.0 g of the samples and shaken for 60 min at 750 rpm. The solution was filtered and diluted with 200 mL of distilled water. The next step was the SPE extraction using OASIS^®^ HLB cartridges (500 mg, 6 mL). OASIS^®^ HLB cartridges were conditioned with 6 mL of methanol and 6 mL of distilled water. The diluted sample was applied to the sorbent at a flow rate of 3 mL min^−1^. The cartridges were vacuum dried, elution was carried out with 10 mL 0.1% acetic acid in methanol. The eluate was evaporated to dryness, then the sample was dissolved in 1 mL 0.1% acetic acid in methanol and filtered through a nylon syringe filter (0.45 µm, 25 mm). The injection volume was 3 µL. The analyses were repeated six times using HPLC-UV-MS/MS (for validation procedure and for analysis of real environmental samples).

### 2.4. Instrumental and Analytical Conditions

The HPLC-UV-MS/MS apparatus consisted of Dionex HPLC system (Dionex Corporation, Sunnyvale, CA, USA) and AB Sciex Q-Trap^®^ 4000 mass spectrometer (Applied Biosystems/MDS SCIEX, Foster City, CA, USA). The chromatograph consisted of an UltiMate 3000 Rapid Separation pump, an UltiMate 3000 thermostat and an UltiMate 3000 autosampler and controlled by the Dionex Chromeleon TM 6.8 software. The columns Kinetex F5 (50 × 2.1 mm; 1.7 µm), Kinetex F5 (100 × 2.1 mm; 1.7 µm), Kinetex C-18 (75 × 2.1 mm; 2.6 µm) from Phenomenex (Torrance, CA, USA), Fortis Diphenyl (50 × 2.1 mm; 1.7 µm) from Frotis Technologies (Niepruszewo, Poland), Poroshell 120 EC-C18 (100 × 3.0 mm; 2.7 µm) and Poroshell 120 EC-C18 (100 × 2.1 mm; 2.7 µm) from Agilient Technologies (Santa Clara, CA, USA) were tested for the chromatographic separation of the analytes. Finally, the separation of the antimicrobials was carried out on a Poroshell 120 EC-C18 (100 × 2.1 mm; 2.7 µm) column using gradient elution.

The mobile phase consisted of mixture of acetonitrile (solvent A) and 0.1% formic acid (FA) in water (solvent B). Initially, the proportion of solvent A in the mobile phase was 5% and after a minute it increased to 25%. After the first minute of the analysis, the proportion of solvent A in the mobile phase increased linearly for 4 min, and its maximum content was 50% (Table 2). The total analysis time was 8 min and the separation of the analytes was 5 min. The flow rate during the analysis from 0.0 to 1.0 min it was 0.8 mL min^−1^, then to 3.5 min it was reduced to 0.5 mL min^−1^, then to 5.0 min was increased to 0.8 mL min^−1^ and till the end of the analysis, the flow rate was constant 0.8 mL min^−1^. The column temperature was 25 °C and the injection volume was 3 µL. The determination was carried out at a wavelength of λ = 250 nm (UV detection was used only during optimization of extraction procedure).

The tandem mass spectrometer (MS/MS) was equipped with electrospray ionization sources (ESI) and controlled by the Analyst 1.4 software. The analytes were performed in multiple reaction monitoring mode (MRM). Four of the five analytes were determined in the positive ionization mode, only CEF was determined in the negative ionization mode. The ion source parameters were as follows: temperature (TEM) = 500 °C, collision gas (CAD) = medium, ion spray voltage (IS) = 4500 V for positive ion mode and −4500 V for negative ion mode, curtain gas (CUR) = 20 psi, ion source gas 1 (GS1) = 60 psi and ion source gas (GS2) = 50 psi. The MRM transitions and optimized MS parameters for each analytes are shown in Table 3.

### 2.5. Method Validation

The newly developed HPLC-UV-MS/MS method for analysis of five antimicrobials in plant tissues and in soil was validated. As part of the validation, the linearity, limit of detection, limit of quantification, accuracy, precision, recovery and the matrix effect were determined. Additionally, the carry-over effect and the stability of the analytes in samples stored in a refrigerator were studied.

Linearity was determined on the basis of the prepared calibration curves for the selected antimicrobials. The calibration curves were in the range of 0.1 ng g^−1^–200 ng g^−1^, three independent series of replicates were prepared for each concentration level (0.1 ng g^−1^; 1 ng g^−1^; 10 ng g^−1^; 30 ng g^−1^; 60 ng g^−1^; 100 ng g^−1^; 150 ng g^−1^; 200 ng g^−1^). The calibration curves were prepared by adding working solutions of the standards at defined concentrations to the matrix solution. The calibration was performed without using internal standard. The matrix solution was prepared by extracting plant material and soil sample not enriched with antimicrobials as described in Section 2.3. Regression equations for each analyte were obtained using the linear regression method, and then the coefficient of determination (R^2^) was determined.

The limit of detection (LOD) and limit of quantification (LOQ) were calculated according to the guideline [44]. Limit of detection was 0.03 ng g^−1^ and limit of quantification was 0.1 ng g^−1^.

In order to determine the accuracy and precision of the method, the plant material and the soil sample were enriched at three levels of analyte concentration: low (LQC = 1 ng g^−1^), medium (MQC = 80 ng g^−1^) and high (HQC = 200 ng g^−1^). The analyses were performed in six replications, and then accuracy, as a relative error, was determined (RE). The precision of the method was determined on the basis of the coefficient of variation (CV).

The effectiveness of the developed extraction procedure was assessed on the basis of the recovery value (R). Samples of plant material and soil samples enriched with antimicrobials at three concentration levels—LQC, MQC, HQC—were prepared and extraction was performed according to the procedure described in Section 2.3. The samples were prepared in triplicate and analyzed in duplicate. The recovery was determined on the basis of the analytes’ peak area measurement and related to the standard solution (enriched matrix solution). The matrix effects (ME) of the plant tissues and the soil were evaluated by comparing the peak area of the compound mixed with real samples that remained after extraction to the peak area of compounds diluted with a mobile phase at equivalent concentrations.

The stabilities of the drugs in plant material and in soil samples were determined at three concentration levels—LQC, MQC, HQC. Stability was evaluated using QC samples kept at temperature 4 °C for 21 days and then analyzed.

Carry-over effect was investigated by injecting three processed blank soil and plant samples subsequently after injection of an upper limit of quantification (ULOQ) sample in three independent runs.

### 2.6. Determination of the Selected Antimicrobials in Environmental Samples

Parsley root samples were purchased from five local stores. Fresh parsley root was first grated and then ground in an electric grinder. The parsley preparation obtained was air-dried to constant mass. Soil samples were collected in areas close to hospitals and medical clinics (urban soils at street and settlement greens). The soil samples were dried and then sieved through a metal sieve with a diameter of 0.6 mm. The developed SLE-SPE extraction procedure, described in Section 2.3, was used for preparation of the parsley root and soil samples.

Due to the low concentration range of antimicrobials in environmental samples, the samples were then analyzed using HPLC-MS/MS in the positive and negative ionization mode. The content of the analytes in the samples was determined using the surface areas and the obtained calibration curves.

## 3. Results and Discussion 

### 3.1. Development of the Chromatography Conditions

The development of the conditions for the chromatographic separation of CIP, ENF, NAL, MET and CEF began with the selection of the chromatographic column. The optimization of the parameters of the chromatographic system was performed on a mixture of antimicrobial standards dissolved in methanol. The chromatographic assay was performed in reverse phase on six chromatographic columns. The columns differed in size, pore diameter and the stationary phase. The stationary phases of silica gel modified with: pentafluorophenyl (Kinetex F5), diphenyl (Fortis Diphenyl) and octadecyl (Kinetex C18, Poroshell 120 EC-C18) groups were checked. The best results were obtained with a Poroshell 120 EC-C18 column (100 × 2.1 mm; 2.7 µm), modified with C18 octadecylsilane groups.

The influence of the composition of the mobile phase on the separation of the analytes’ signals was checked for solvents such as water, acetonitrile, methanol and 0.1% FA in water. The elution was carried out in a gradient system. The best result was obtained with the system of (A) acetonitrile and (B) 0.1% FA in water. The optimized gradient elution program is shown in Table 2. The maximum acetonitrile content in the mobile phase was 50%. The use of methanol instead of acetonitrile resulted in overlapping signals and deterioration of the symmetry. Changing the flow rate from 3.5 to 5.0 min from 0.8 mL min^−1^ to 0.5 mL min^−1^ allowed the separation of ENF signals from CIP, with a similar chemical structure. The optimal temperature of the column thermosetting was 25 °C, increasing the temperature to 30 °C caused a decrease in the selectivity of the method by visualizing the matrix signals. At lower temperatures, the intensity of the analyte signals decreased.

A spectrophotometric detector (UV) was used for the analysis of antimicrobials only during optimization of the extraction procedures. The intensity of the signals of the analytes tested at wavelength of 220, 230, 250 and 310 nm was the highest. Finally, the analysis was carried out at a wavelength of λ = 250 nm, at which most of the antimicrobials had their absorption maximum. At lower wavelengths of light (220 and 230 nm) an increase in the intensity of the matrix signals was observed. At λ = 310 nm, a decrease in signal intensity was observed as compared to the signals recorded at λ = 250 nm.

**Figure 1:**
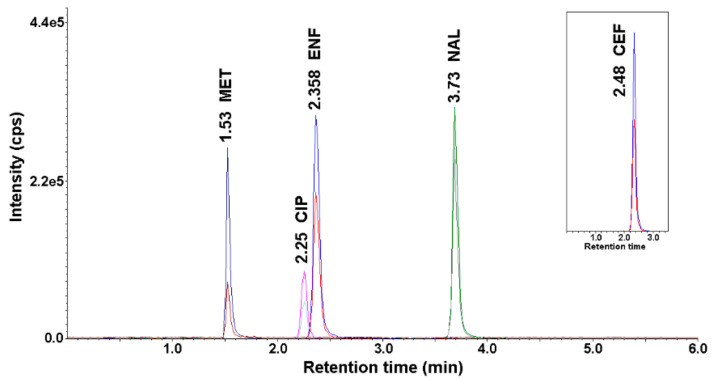
Chromatogram of antimicrobials standard solution (concentration 50 ng mL^−1^) after application of the developed high-performance liquid chromatography coupled with an ultraviolet (UV) detector and a tandem mass spectrometer (HPLC-UV-MS/MS) method.

The optimal conditions for the chromatographic determination using the HPLC-UV-MS/MS method are described in Section 2.4. Figure 1 shows a representative chromatogram of a mixture of antimicrobial standards recorded under the described HPLC-UV-MS/MS conditions.

### 3.2. Development of the Extraction Procedure

The extraction procedure consisted of two steps—liquid-solid extraction (SLE) and pre-concentration and purification of the sample using solid-phase extraction (SPE). Blank samples (parsley root and soil) were selected as the research material, which were pre-prepared and enriched with a mixture of antimicrobials of a specific concentration.

**Figure 2:**
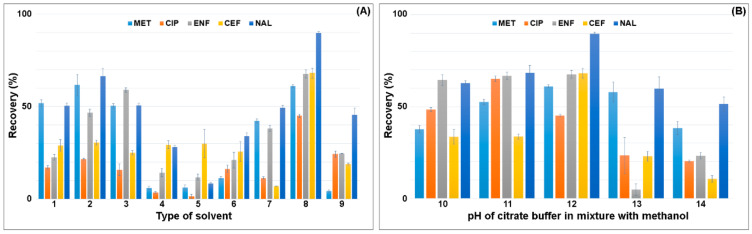
Effect of the (**A**) type of solvent on recovery of antimicrobials (1: methanol; 2: methanol:acetonitrile (1:1; *v*/*v*); 3: methanol:0.1 M HCl (1:1; *v*/*v*); 4: acetonitrile:acetone (1:1; *v*/*v*); 5: acetonitrile:ethyl acetate (1:1; *v*/*v*); 6: acetonitrile:0.01 M HCl (1:1; *v*/*v*); 7: 2% acetic acid in methanol:acetonitrile (1:1; *v*/*v*); 8: citrate buffer (pH = 4):methanol (1:1; *v*/*v*); 9: citrate buffer (pH = 4):acetonitrile (1:1; *v*/*v*)). (**B**) pH of citrate buffer in mixture with methanol (10: citrate buffer (pH = 2):methanol (1:1; *v*/*v*); 11: citrate buffer (pH = 3):methanol (1:1; *v*/*v*); 12: citrate buffer (pH = 4):methanol (1:1; *v*/*v*); 13: citrate buffer (pH = 5):methanol (1:1; *v*/*v*); 14: citrate buffer (pH = 6):methanol (1:1; *v*/*v*)).

The optimization procedure was started with the selection of the type of solvent and its volume in the SLE extraction. Based on the literature and information on the physical and chemical properties of the compounds, the solvents to be used to extract pharmaceuticals from plant material samples and soil were selected. Mixtures of methanol, acetonitrile, acetone, ethyl acetate and citrate buffer were used for the extraction under acidic to neutral pH conditions. The recovery of the selected solvents in SLE extraction is shown in Figure 2.

The lowest recovery of the analytes, 5–30%, was obtained for mixtures of acetonitrile with acetone and with ethyl acetate. Extraction with pure methanol gave recoveries ranging from 50–60%. The addition of acetonitrile to methanol in a 1:1 volume ratio increased the recovery of MET and NAL by 10% and ENF by 25% compared to pure methanol. The use of acidified mixtures of acetonitrile:0.01 M HCl in water and 2% acetic acid in methanol:acetonitrile (1:1; *v*/*v*) resulted in lower recovery of the analytes, compared to extraction in pure solvents. The highest recovery was obtained for the mixture of citrate buffer (pH = 4):methanol (1:1; *v*/*v*) 45% (CIP)—90% (NAL). The conversion of methanol to acetonitrile caused the extraction yield to drop by 50% due to the lower polarity of the mixture. The pH of the citrate buffer significantly affected the recovery value. The citrate buffer was checked in the range of pH 2–6. The highest recovery was obtained for the citrate buffer with pH 4. The use of the citrate buffer pH 3 resulted in an increase in CIP recovery by 20% and a decrease in CEF and NAL recovery by 35% and 20%, respectively. A further reduction in pH resulted in a decrease in the extraction efficiency of antimicrobials due to their low stability at acidic pH. The lowest recovery was obtained for the mixture of citrate buffer pH = 6:methanol (1:1; *v*/*v*) and ranged from 10–50%.

**Figure 3:**
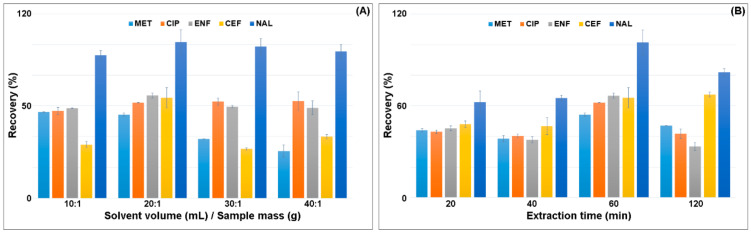
Effect of the (**A**) ratio of the mass of the sample to the volume of the solvent and (**B**) extraction time on the recovery of antimicrobials.

In the next step, the ratio of the sample weight to the solvent volume was selected. The highest recovery of analytes was obtained for the ratio of 1.0 g of sample:20 mL of solvent (Figure 3A). The extraction was performed one, two or three times. Single extraction with 20 mL of solvent gave the highest recovery of all the analytes. Increasing the extraction ratio resulted in the extraction of more matrix components and decreased the recovery of MET, CEF and NAL by 20–40% (Figure 3A). The increase in the solvent volume resulted in a slight increase in CIP recovery and a decrease in CEF recovery by 20%. Reduction in the solvent volume also decreased the CEF recovery.

The extraction time significantly influenced the recovery of the analytes from the plant matrix. Shaking was performed for 20, 40, 60 and 120 min (Figure 3B). The highest recovery was obtained for the time of 60 min. Increasing the shaking time to 120 min reduced the recovery of analytes. Shaking was carried out at two rotational speeds of 750 rpm and 900 rpm. Increasing the agitation intensity decreased the recovery of the analytes by 10–25%.

As an alternative to shaking, the effect of ultrasound on the extraction yield was checked. The samples were extracted in a water bath for 60 min. The recovery of the analytes obtained with the UAE technique was lower than that for the traditional SLE and ranged from 15% to 60%. Most likely, it was caused by the lack of stability of antimicrobials under the conditions of ultrasound treatment and, consequently, their degradation.

The purification and concentration of the sample was performed by SPE extraction using antimicrobials. The SPE extraction efficiency of the selected analytes with the use of standards allows for recovery in the range of 80–100%. When the SLE-SPE method was combined, the recovery of the analytes ranged from 55% (MET) to 100% (NAL). An optimized SLE-SPE procedure for the extraction of antimicrobials from solid samples is described in Section 2.3.

### 3.3. Method Validation

The developed method of isolating five antimicrobials from plant and soil samples and their determination with the HPLC-UV-MS/MS method has been validated. For this purpose, its linearity, limit of detection (LOD), limit of quantification (LOQ), accuracy, precision, selectivity and recovery were determined. The validation was performed on blank extracts enriched with antimicrobials. The enriched samples were prepared in triplicate and analyzed in duplicate. The calibration curves were prepared by measuring the area under the analytes signals, and then, using the linear regression function, the equations of the calibration curves for the antimicrobials were obtained.

The linearity of the developed HPLC-UV-MS/MS method was in the range of 0.1–200 ng g^−1^. The values of the correlation coefficient (R^2^) of the obtained curves were higher than 0.99 (Table 4). The limit of quantification (LOQ) was taken as the lowest concentration value on the calibration curve (0.1 ng g^−1^), and the limit of detection designated as 1/3 of the LOQ was 0.03 ng g^−1^. The accuracy and precision of the method was determined on the basis of the analysis of blank extracts enriched with antimicrobials at three concentration levels: low (LQC = 1 ng g^−1^), medium (MQC = 80 ng g^−1^) and high (QC = 160 ng g^−1^). The samples were prepared in triplicate and analyzed in duplicate. The accuracy was assessed on the basis of the degree of agreement between the given and measured concentration value of the analyte standards. The accuracy was determined on the basis of the relative error (RE), for which the obtained values were between −5.78% and 6.18% for each concentration level (Table 4). Precision was established on the basis of the consistency of the results determined from the relative standard deviation (RSD), where RSD <6%, both for soil samples and parsley root samples (Table 4). In order to determine the repeatability of the method, the analyses were performed on different days and at different times. The matrix effect values in soil samples and parsley root samples were all between 1.96% to 6.89% for analysed compounds at different QC levels (Table 4). For analyzed drugs the carry-over detected in the first blank sample was between 1.25% and 3.15% of the response detected in a LOQ soil and plant samples. Therefore, the carry-over effect was found to be negligible from previous concentrated samples. Moreover, it was found, that all compounds were found to be stable in soil and plant samples stored in refrigerator during 21 days (accuracy ranged from −5.13% to 4.04%, the precision ranged from 1.17% to 6.21%).

The analyte recovery for the optimized SLE-SPE sample preparation procedure was also assessed at three concentration levels. The recoveries of the five selected antimicrobials obtained ranged from 55.9–103%. The lowest recovery was obtained for CEF and CIP (about 60%). The result obtained is similar to that obtained in the literature [23,26,45,46] and is satisfactory. The selectivity of the method was determined by comparing the retention times of the analytes dissolved in the matrix against the standard dissolved in methanol and the non-enriched matrix solution. The signals were well separated and no matrix interference was observed after the analysis of the extracts of soil samples and parsley root samples.

### 3.4. Determination of Selected Antimicrobials in Environmental Samples

**Figure 4:**
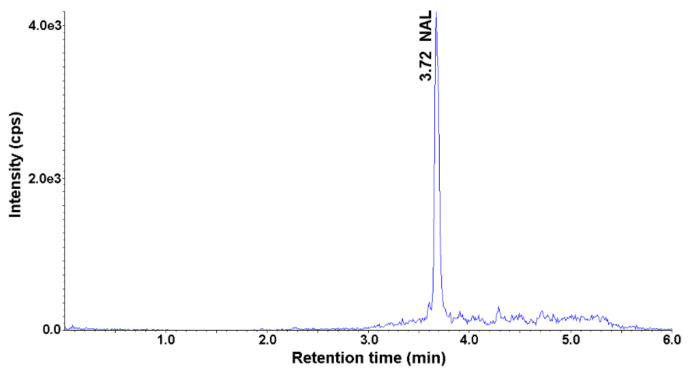
Representative MRM chromatogram of the parsley root sample obtained after application of SLE-SPE-HPLC-UV-MS/MS procedure.

The content of the selected antimicrobials was analyzed in five soil samples and five parsley root samples. The parsley root was bought in local stores, while the soil was collected at different points in Gliwice, Poland. Soil and parsley samples were prepared according to the described SLE-SPE procedure and analyzed using HPLC-UV-MS/MS. None of the selected analytes was detected in the soil samples. NAL was detected in the parsley root in three out of five parsley root samples. The highest measured concentration of NAL in the root was 0.72 ng g^−1^ (Figure 4). This confirms the ability of plants to absorb antimicrobials from the environment and accumulate in tissues.

Preparations containing NAL were withdrawn from the European market in 2019 by the European Medicines Agency. Nalidixic acid is rarely used due to the high dose required to produce a therapeutic effect. Moreover, reports have shown that the bacterial strains present in the environment are resistant to the action of fluoroquinolone antimicrobials, with NAL resistance genes being most commonly detected (74–95%) [45,46,47,48,49]. There is no information in the literature on the bioaccumulation of NAL in plant tissues. NAL is found mainly in the meat of farm animals [50,51] and soil [52]. It can be assumed that the source of NAL in parsley root was soil fertilized with contaminated animal manure, because quinolones may be present in the soil even several months after fertilization [53].

## 4. Conclusions

In this work, a new procedure for the extraction and determination of selected pharmaceuticals from plant tissues and soil samples was developed and applied for the analysis of real, environmental samples. A two-step procedure for extracting analytes from environmental samples was optimized using solid-liquid extraction combined with sample purification on SPE. The procedure was validated and its recovery value was comparable to the methods described in the literature. The developed procedure was used to analyze soil samples and parsley root. Nalidixic acid was detected in three out of five parsley root preparations. This confirms that plants are able to accumulate pharmaceuticals in plant tissues. The obtained results are a novelty because no information on the bioaccumulation of NAL in plant tissues can be found in the literature.

Moreover, as a conclusion, a new, safer, environmentally friendly, faster method for extraction of selected emerging contaminants was developed during this study, which can be successfully applied to environmental matrices.

## Figures and Tables

**Table 1:** Physicochemical properties of selected antimicrobials.

Analyte	Structure	Molar Mass (g mol^−1^)	pK_a_	logP	Ref.
CIP	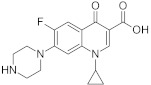	331.38	3.64	1.32	[38,39]
ENF	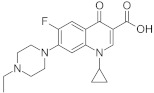	359.39	0.70	6.0 8.8	[20]
NAL	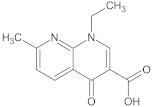	232.24	6.41	1.86	[40]
CEF	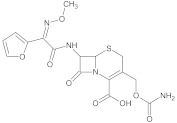	424.39	2.11	−0.90	[41,42]
MET	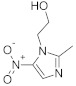	171.16	0.02	2.40	[43]

CIP—ciprofloxacin, ENF—enrofloxacin, NAL—nalidixic acid, CEF—cefuroxime, MET—metronidazole.

**Table 2:** Selected gradient elution program.

Time (min)	A (%)	B (%)	Flow Rate (mL min^−1^)
0.0	5	95	0.8
1.0	25	75	0.8
3.5	45	55	0.5
5.0	50	50	0.8
6.0	10	90	0.8
6.1	5	95	0.8
8.0	5	95	0.8

**Table 3:** Multiple reaction monitoring (MRM) conditions for the analysis of the selected antimicrobials.

Analyte	R_T_ (min)	Q_1_	Q_3_	Time (ms)	DP (V)	CE (V)	CPX (V)	EP (V)
MET	1.53	172.1	**128.1** 82.1	50	76	21 33	8 6	7
CIP	2.25	332.0	**288.0** 314.0	50	86	25 29	8 8	7
ENF	2.35	360.8	**316.2** 245.2	50	101	29 27	8 10	7
CEF	2.48	423.0	**207.0** 318.1	250	−45	−20 −12	−5 −9	−10
NAL	3.73	233.0	**215.2** 187.2	50	56	21 35	12 14	7

Q_1_—precursor ion; Q_3_—fragment ion *(quantitative ion—bold*; DP- declustering potential; CE—collision energy, CXP—cell exit potential; EP—entrance potential; R_T_—retention time. MET—metronidazole, CIP—ciprofloxacin, ENF—enrofloxacin, CEF—cefuroxime, NAL—nalidixic acid.

**Table 4:** The analytical parameters of the developed solid-liquid extraction (SLE)-solid phase extraction (SPE)-HPLC-UV-MS/MS procedure.

Analyte	Linear Range (ng g^−1^)	R^2 a^	LOD ^b^ (ng g^−1^)	LOQ ^c^ (ng g^−1^)	Concentration (ng g^−1^)	Intra-Day		Inter-Day		ME ^f^ (%)	Recovery ± SD (%)
						CV (%) ^d^	RE (%)^e^	CV (%) ^d^	RE (%) ^e^		
**Soil samples/Parsley root**											
MET	0.1–200				1	4.17	−5.78	5.18	−6.98	4.89	65.8 ± 4.74
		0.9924	0.03	0.1	80	3.45	−4.13	3.97	−5.72	3.71	67.3 ± 5.17
					160	2.15	−2.47	3.58	−3.56	3.12	67.9 ± 3.63
CIP	0.1–200				1	4.78	−4.32	6.19	−7.12	5.17	55.9 ± 3.56
		0.9930	0.03	0.1	80	3.98	−3.69	5.78	−6.37	4.69	57.6 ± 2.87
					160	3.01	−1.78	4.13	−2.53	2.43	60.8 ± 4.11
ENF	0.1–200				1	5.85	−5.47	7.47	−7.17	2.15	81.4 ± 5.42
		0.9963	0.03	0.1	80	5.41	−3.82	6.93	−5.42	1.96	84.6 ± 5.30
					160	4.18	−3.07	3.78	−4.19	2.14	87.6 ± 5.03
CEF	0.1–200				1	4.41	4.92	5.74	5.36	6.89	62.5 ± 4.78
		0.9934	0.03	0.1	80	3.74	3.47	4.12	4.97	5.93	64.0 ± 3.17
					160	1.97	2.15	4.08	3.78	5.05	63.7 ± 2.98
	0.1–200				1	5.25	6.18	6.24	7.59	5.14	90.8 ± 3.82
NAL		0.9956	0.03	0.1	80	4.74	4.87	4.98	5.47	3.96	99.1 ± 2.29
					160	3.16	4.14	3.76	3.69	2.79	103 ± 1.58

^a^ R^2^—correlation coefficient, ^b^ LOD—limit of detection, ^c^ LOQ—limit of quantification, ^d^ CV—coefficient of variation, ^e^ RE —relative error (RE = (measure value—theoretical value/theoretical value)·100%), ^f^ ME—matrix effect. MET—metronidazole, CIP—ciprofloxacin, ENF—enrofloxacin, CEF—cefuroxime, NAL—nalidixic acid.
